# Supplementary material for: A Novel LRRK2 Variant p.G2294R in the WD40 Domain Identified in Familial Parkinson’s Disease Affects LRRK2 Protein Levels
Source: Int J Mol Sci. 2021 Apr 2;22(7):3708. doi: 10.3390/ijms22073708 (PMC8038167; doi:10.3390/ijms22073708)
Supplement: Supplementary file 1 [file ijms-22-03708-s001.zip › Supplemental files/Supplementary Materials 210329.pdf]

Supplementary figure legends

Supplementary Figure 1.

(**A**) Location of the G2294 residue in the dimerized LRRK2 WD40 repeat domain. Each WD40 repeat domain depicted by the ribbon model is colored in green and cyan. The G2294 residue in the red circle is colored in yellow in the left domain. (**B**) G2294 is located in the loop structure between ß strand 13 (ß13) and ß strand 14 (ß14). The G2294 side chain is depicted by the stick model. (**C**) The R2294 side chain is depicted by the stick model. (**D**) Amino acid sequence alignment containing ß13-ß16 of LRRK2 WD40 among the indicated species. The G2294 residue in humans is indicated by a pink arrow. The putative secondary structures are depicted on the upper side, based on previous studies [15]. The crystal structure of the dimerized LRRK2 WD40 repeat domain (PDB ID: 6dlo) was created using PyMOL version 2.0.7 (**A-C**) [15].

Supplementary Figure 2.

(**A**) LRRK2 protein levels in Flp-In 293 T-REx cells harboring a copy of the FLAG-LRRK2 transgene were analyzed by western blotting. To show the endogenous LRRK2 levels in a mock control (-), western blotting with anti-LRRK2 was also performed. Graphs (bars represent mean) represent relative FLAG-LRRK2 levels normalized to β-actin and the ratio of pRab10 to total Rab10. n = 3 biological replicates. Student's *t*-test. β-Actin served as a loading control. (**B**) HEK293T cells expressing FLAG-LRRK2 WT or p.G2294R were treated with DMSO as a mock control, 10 μM MG-132 (proteasome inhibitor), or 100 nM bafilomycin A1 (autophagy inhibitor) for 8 hours. n = 3 biological replicates. Dunnett's test *vs.* mock control. Inhibition of autophagy flux was validated by the increased LC3-II/LC3-I ratio.

Supplementary Figure 3.

Bright field images of macrophages differentiated from human monocytes treated with

(+) or without (-) zymosan. Scale bars, 20 μm.

Supplementary Figure 4.

Individual images of the left plot in Figure 4C. Images were taken using a definite parameter and setting. Bars = 50 μm.

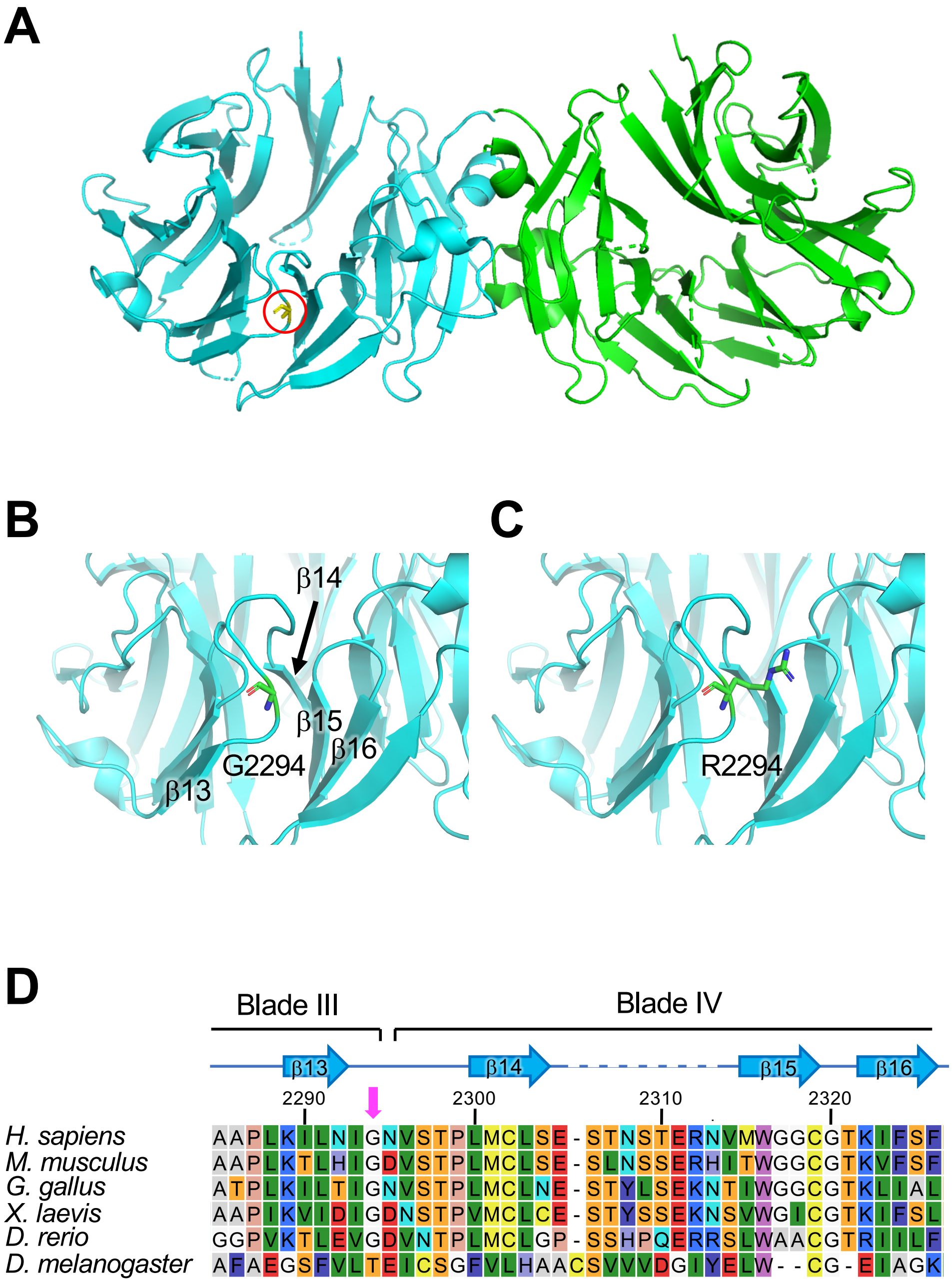
A
B
β14
β15
β16
G2294
β13
C
R2294
D
Blade III
Blade IV
β13
β14
β15
β16
2290
2300
2310
2320
H. sapiens AAPLKILNIGNVSTPLMCLSE-STNSTERNVMWGGCGTKIFSF
M. musculus AAPLKTLHIGDVSTPLMCLSE-SLNSSERHITWGGCGTKVFSF
G. gallus ATPLKILTIGNVSTPLMCLNE-STYLSEKNTIWGGCGTKLIAL
X. laevis AAPIKVIDIGDNSTPVMCLCE-STYSSEKNSVWGICGTKIFSL
D. rerio GGPVKTLEVGDVNTPLMCLGP-SSHPQERRSLWAACGTRIILF
D. melanogaster AFAEGSFVLTEICSGFVLHAACSVVVDGIYELW--CG-EIAGK

Supplementary Figure 2 (Ogata *et al.*)

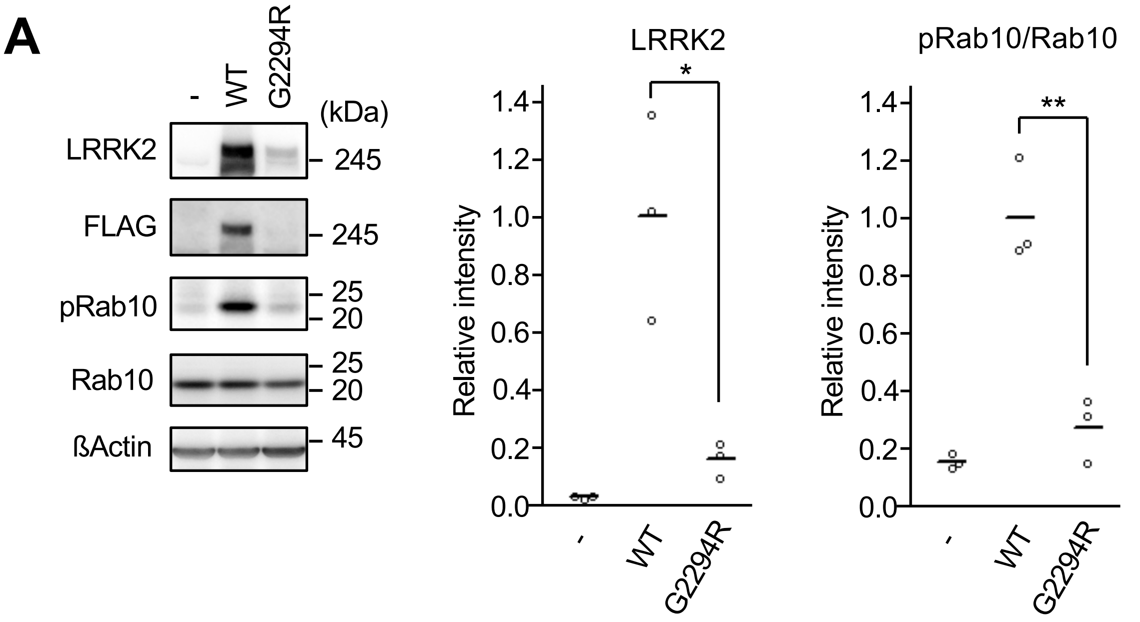

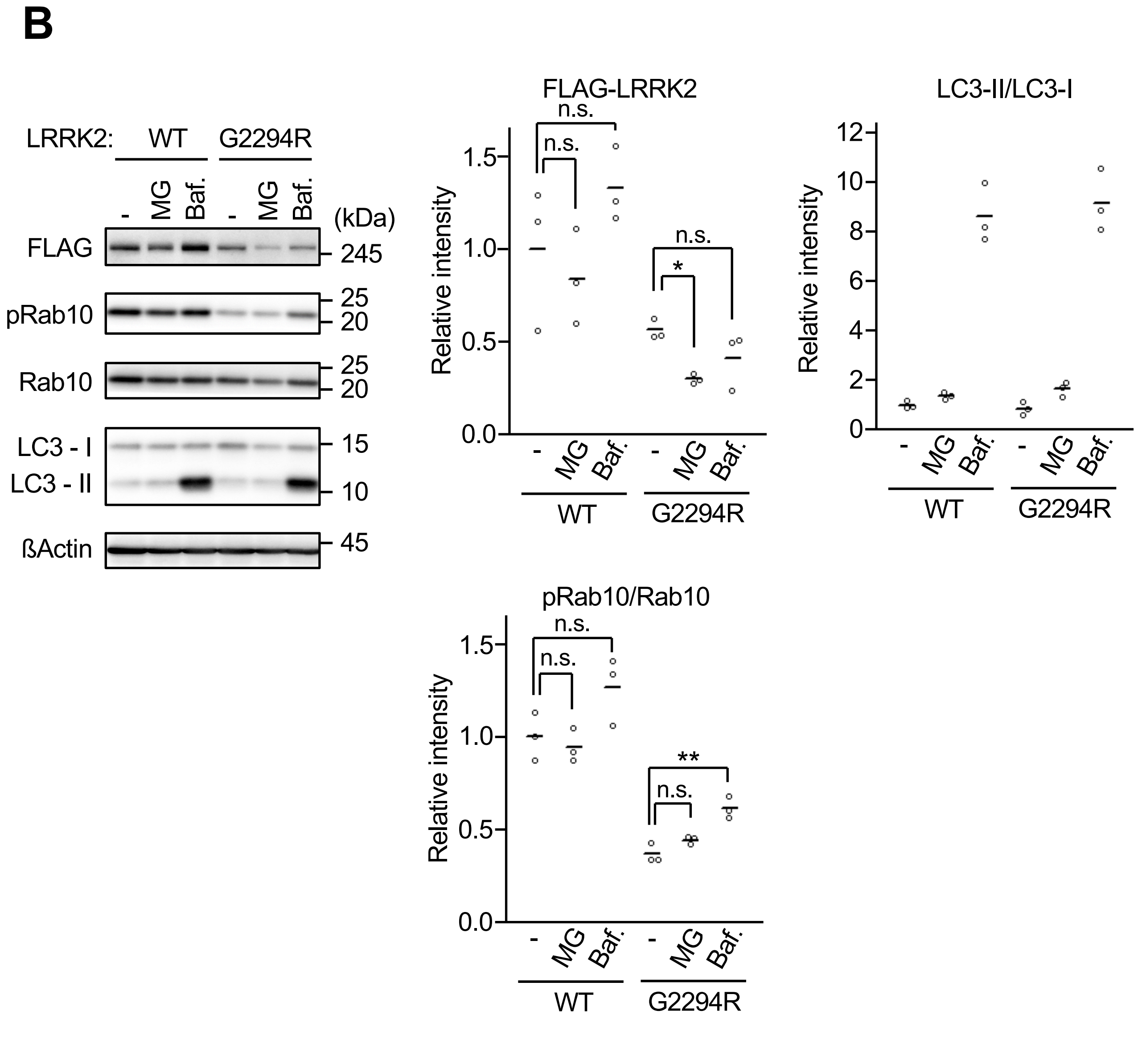

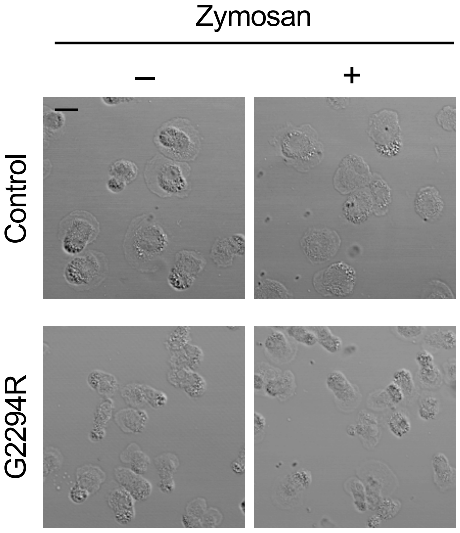

Zymosan
–
+
Control
G2294R

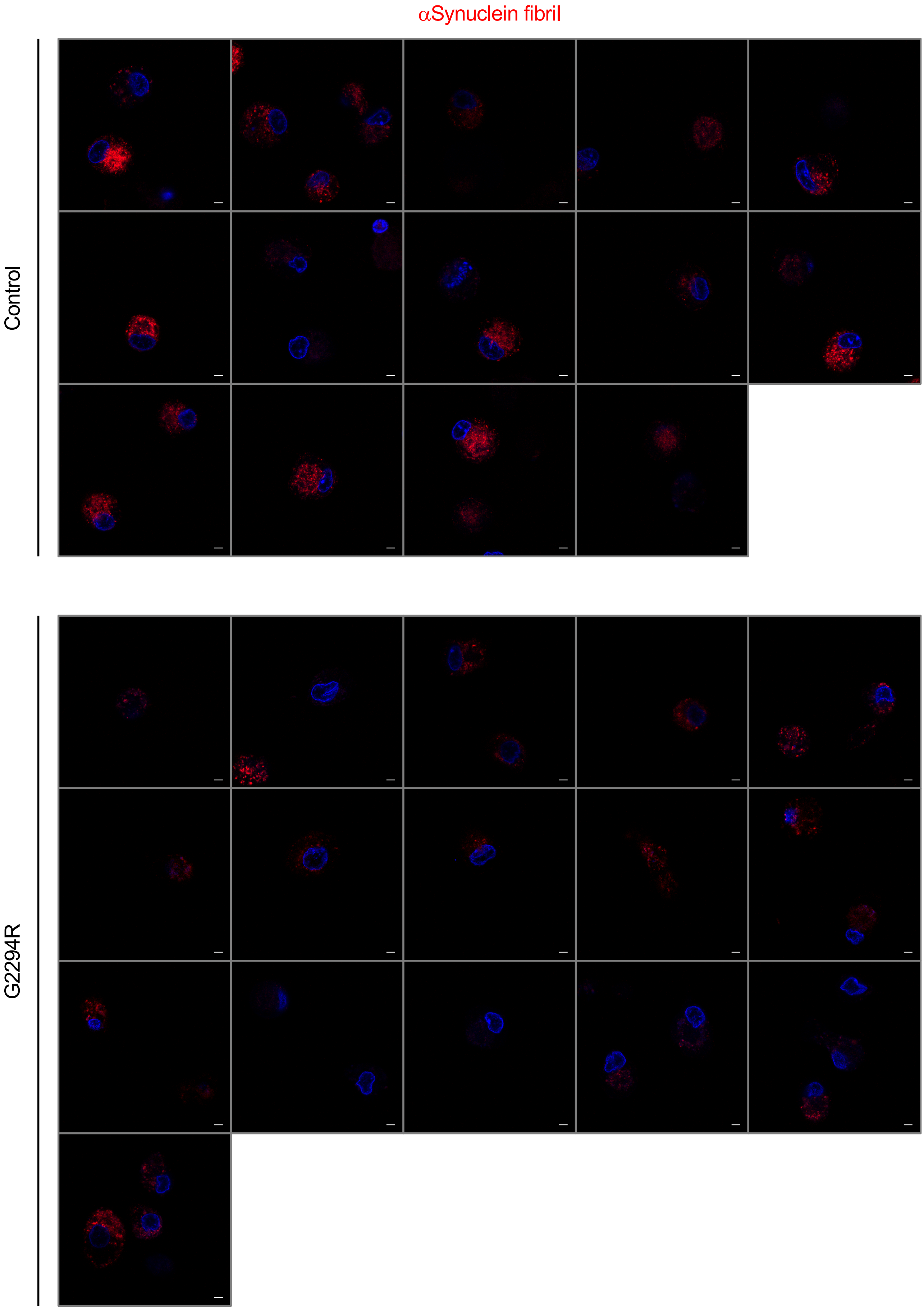
αSynuclein fibril
Control
G2294R
