# Supplementary material for: A Novel LRRK2 Variant p.G2294R in the WD40 Domain Identified in Familial Parkinson’s Disease Affects LRRK2 Protein Levels
Source: Int J Mol Sci. 2021 Apr 2;22(7):3708. doi: 10.3390/ijms22073708 (PMC8038167; doi:10.3390/ijms22073708)

For Figure 2A

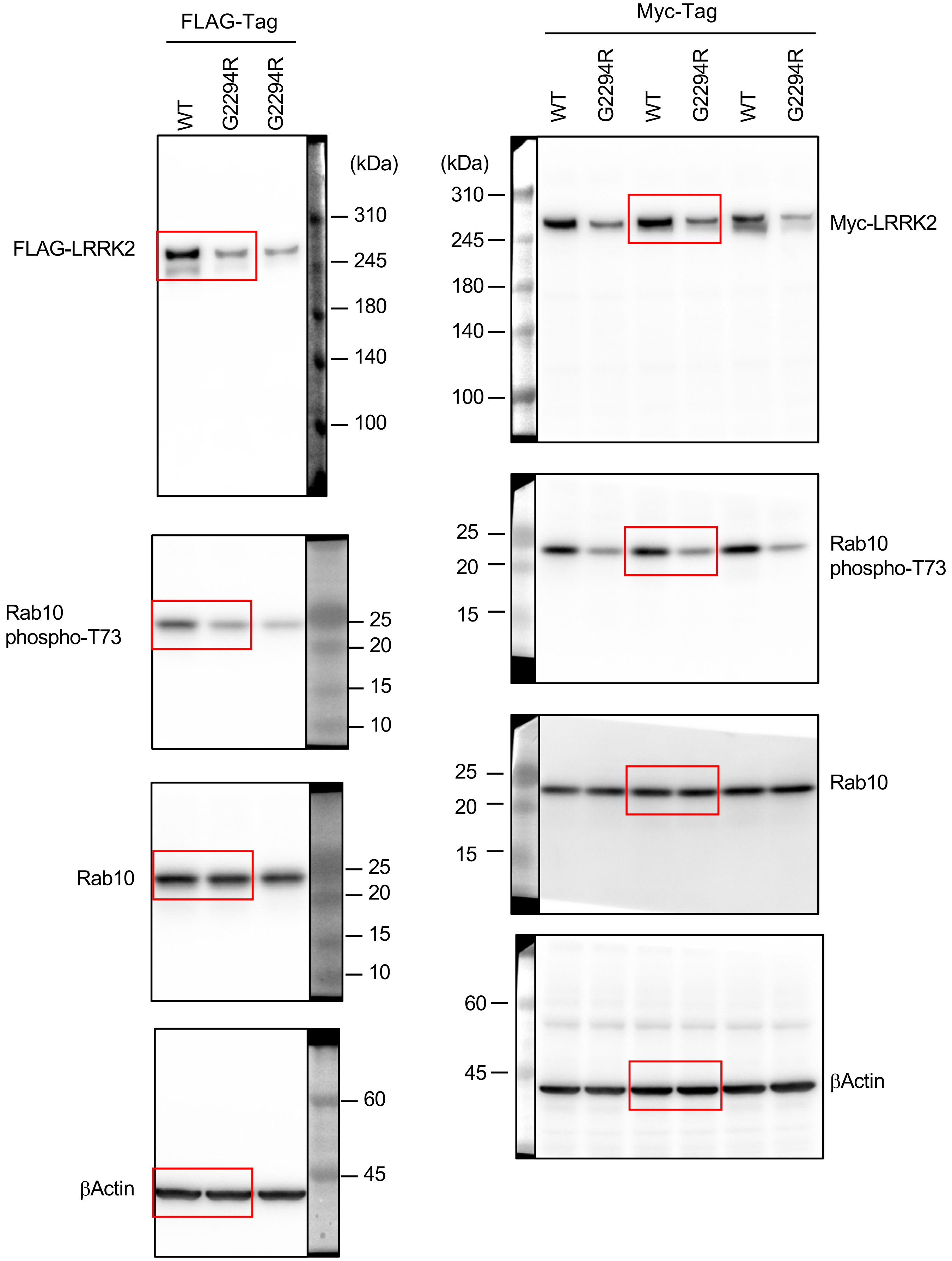

For Figure 2B

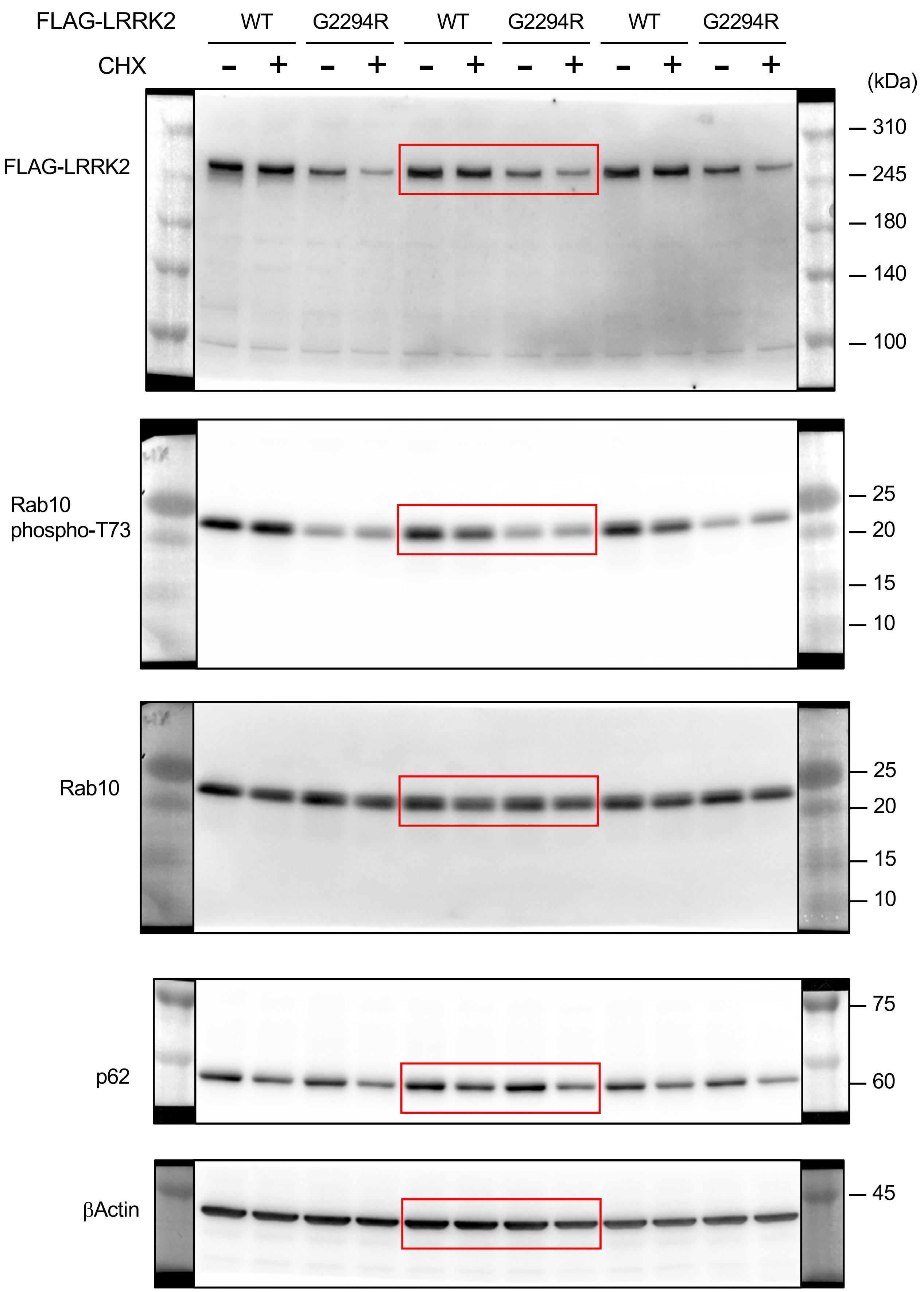

For Figure 2C

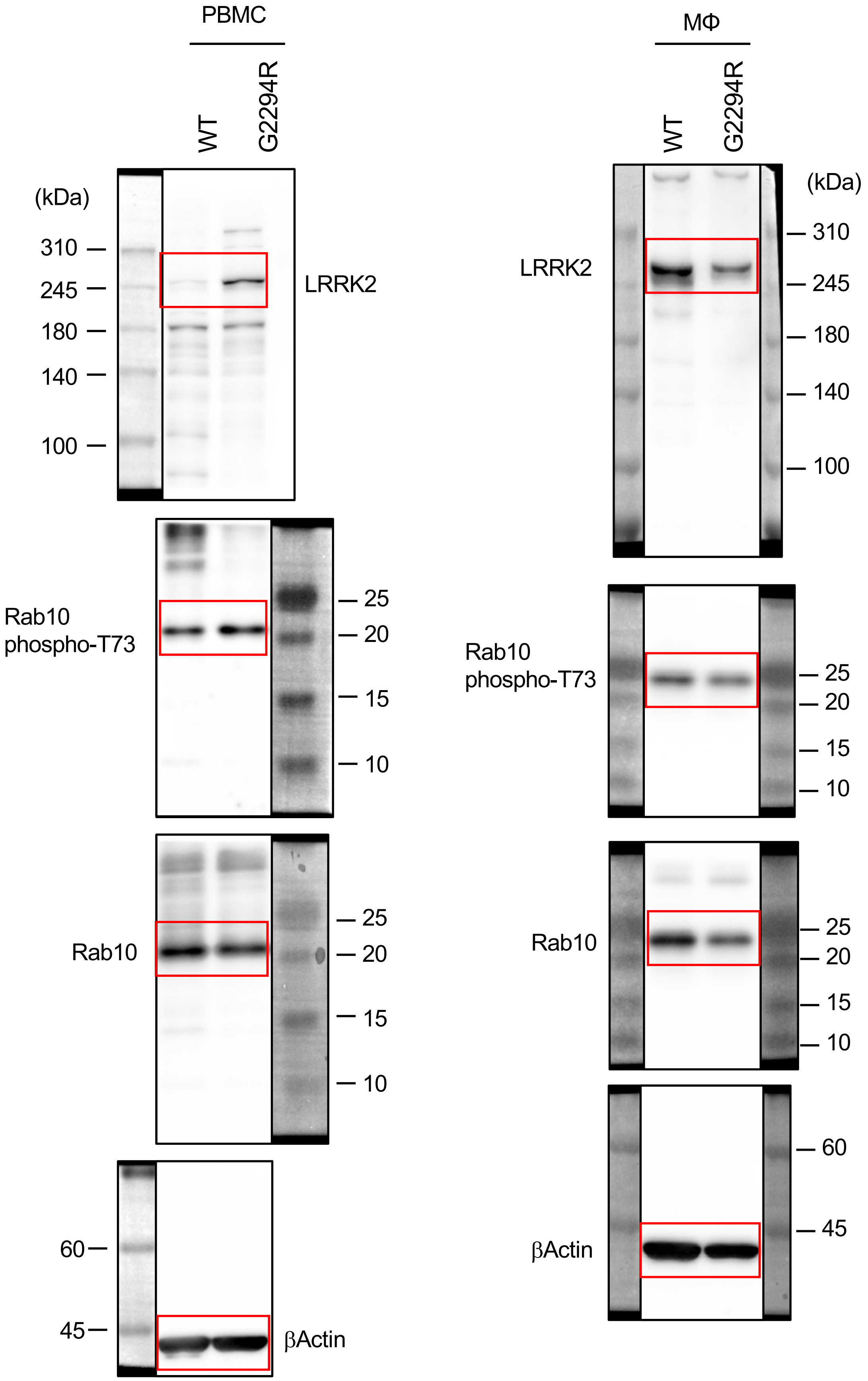

For Figure 2A and C

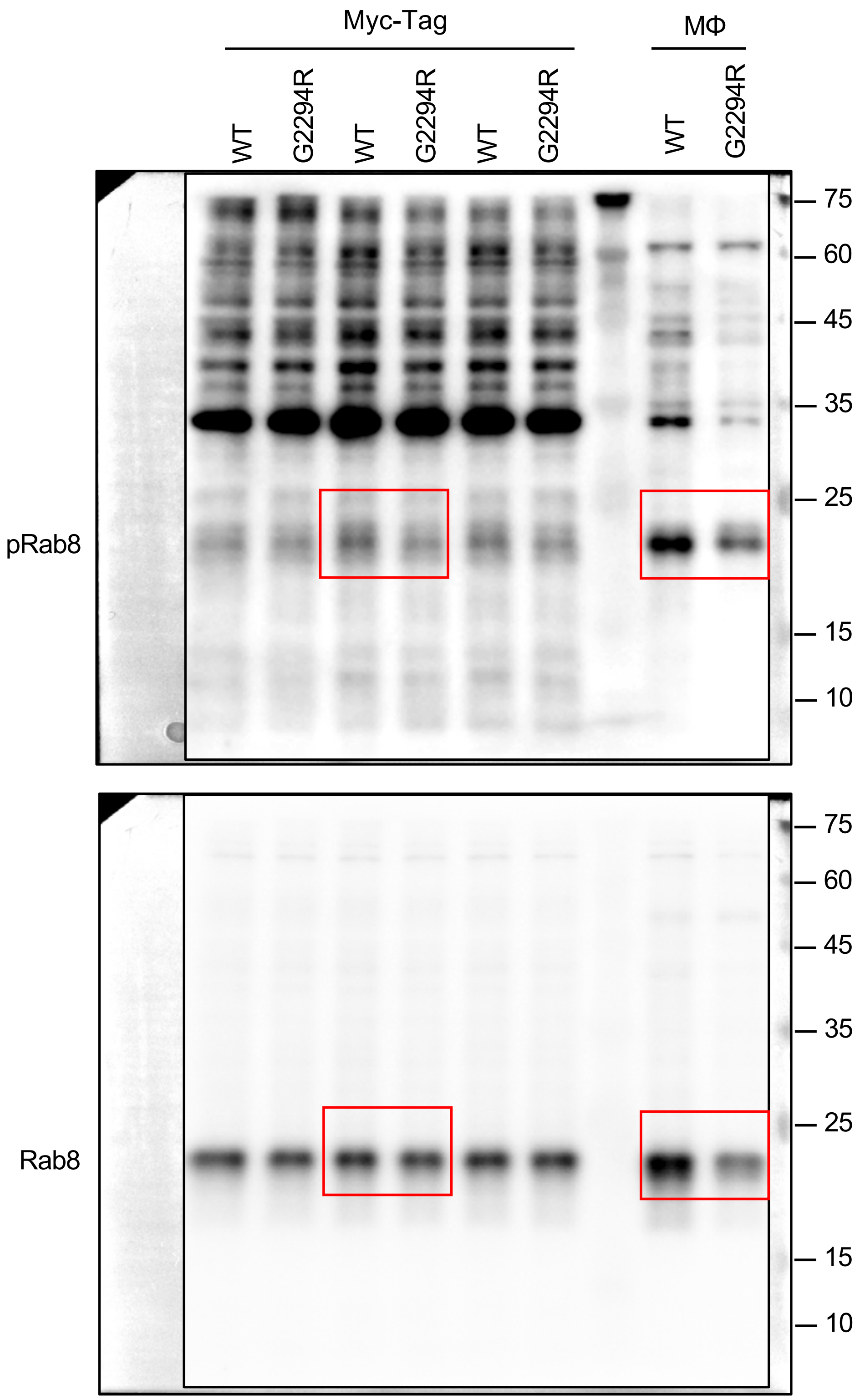

For Supplemental Figure 2A

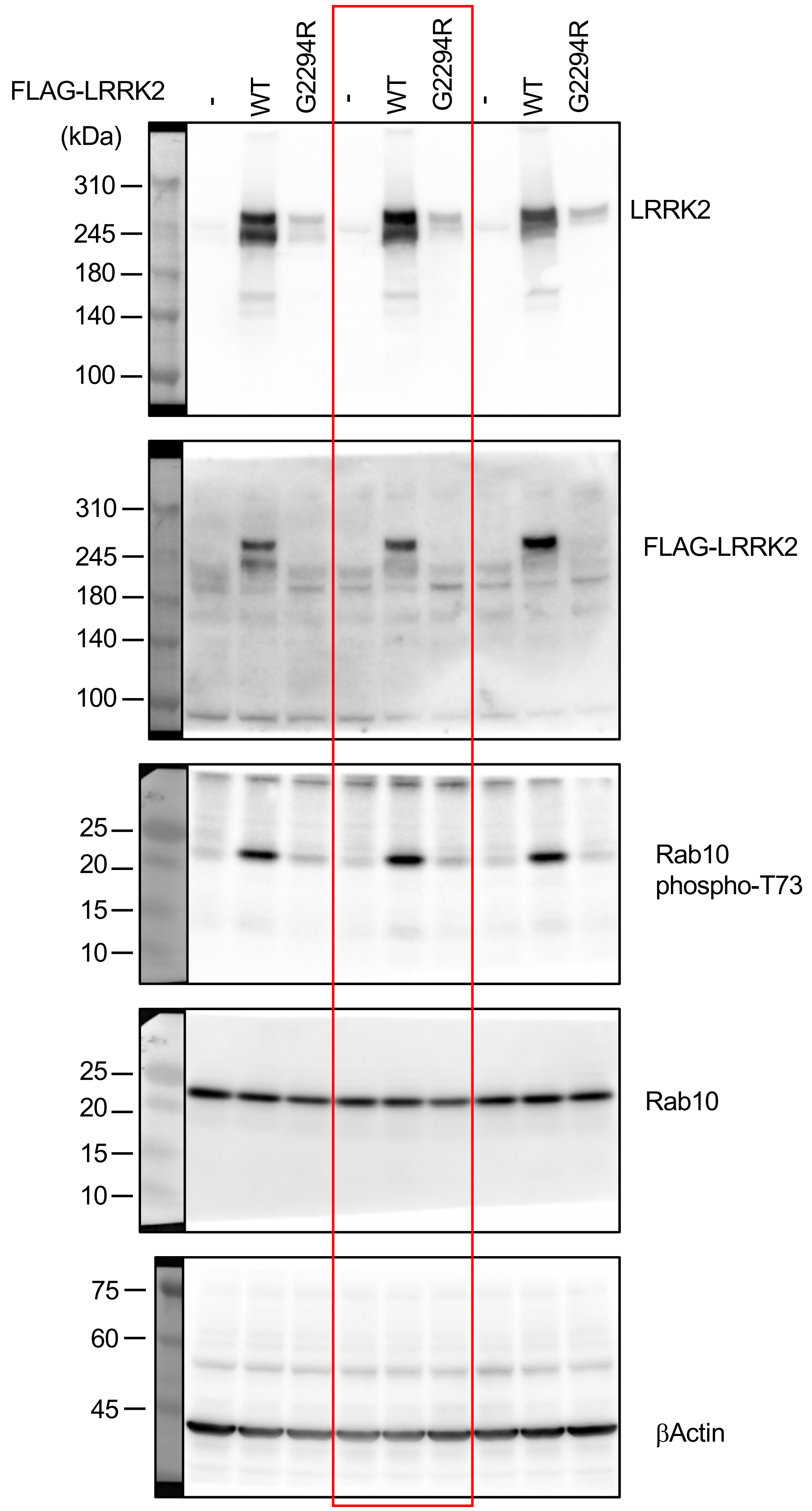

For Supplemental Figure 2B

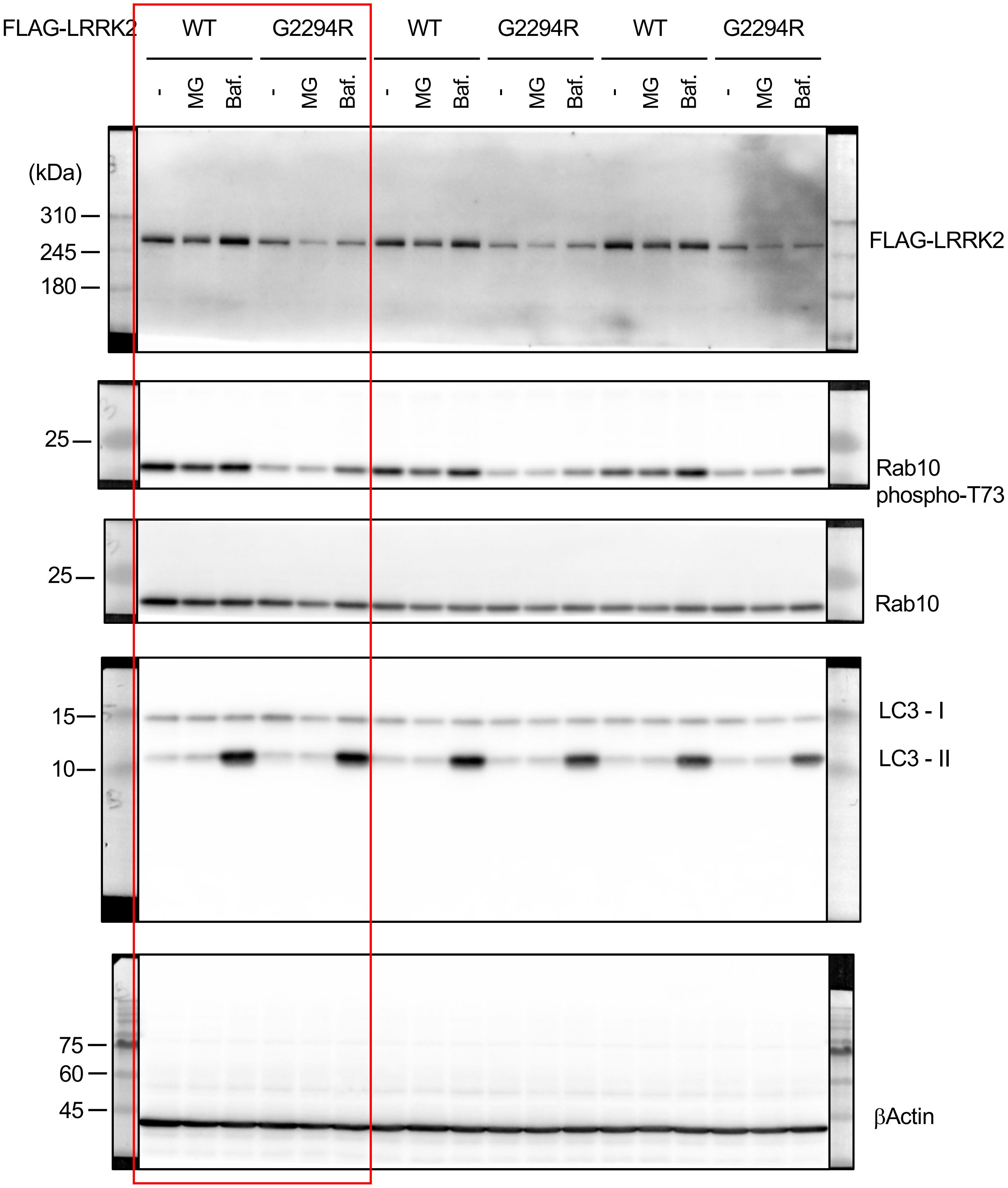

Supplement: Supplementary file 1 [file ijms-22-03708-s001.zip › Supplemental files/Uncropped western blot data.pdf]
